# CeO_2_-Azacrown Conjugate as a Nanoplatform for Combined Radiopharmaceuticals

**DOI:** 10.3390/nano12244484

**Published:** 2022-12-18

**Authors:** Sofia Khabirova, Gleb Aleshin, Tatiana Plakhova, Anastasia Zubenko, Anna Shchukina, Olga Fedorova, Aleksey Averin, Ekaterina Belova, Elena Bazarkina, Kristina Kvashnina, Stepan Kalmykov

**Affiliations:** 1 Department of Chemistry, Lomonosov Moscow State University, Leninskie Gory, 1/3, 119991 Moscow, Russia; 2 N. Nesmeyanov Institute of Organoelement Compounds of Russian Academy of Sciences, Vavilova, 28, GSP-1, 119991 Moscow, Russia; 3 Frumkin Institute of Physical Chemistry and Electrochemistry Russian Academy of Sciences, Leninskiy Ave. 31b4, 119991 Moscow, Russia; 4 The Rossendorf Beamline at ESRF—The European Synchrotron, CS40220, CEDEX 9, 38043 Grenoble, France; 5 Helmholtz Zentrum Dresden-Rossendorf (HZDR), Institute of Resource Ecology, P.O. Box 510119, 01314 Dresden, Germany

**Keywords:** nanoceria, surface functionalization, radiolabeled nanoparticles, bismuth, radiopharmaceuticals

## Abstract

This study is one of the first attempts to assess CeO_2_ nanoparticles as a nanoplatform for radiopharmaceuticals with radionuclides. The process of functionalization using a bifunctional azacrown ligand is described, and the resulting conjugates are characterized by IR and Raman spectroscopy. Their complexes with ^207^Bi show a high stability in medically relevant media, thus encouraging the further study of these conjugates in vivo as potential combined radiopharmaceuticals.

## 1. Introduction

Diagnostics incorporating labeled nanoparticles (NPs) can be used for the early detection, characterization, and staging of diseases, as well as for effective radionuclide therapy [1]. The small size of nanoparticles allows them to pass through cell membranes and deliver the radiopharmaceutical closer to the nucleus of the tumor cell, thus reducing the effect of ionizing radiation on healthy organs and tissues [2]. In addition, nanoparticles are often effective vectors for drug delivery [3], whereby the pharmacokinetic and pharmacodynamic properties of the nanoparticles can be optimized by modifying their surface.

Cerium dioxide (or ceria) nanoparticles can be used for various biomedical purposes due to their unique properties. The toxicity of cerium dioxide nanoparticles has been studied previously both in vitro and in vivo [4]. It was shown that cerium dioxide nanoparticles stabilized by biocompatible surfactants (citric acid, dextran, PVP, polyacrylate, PEG) are not toxic to cells even in high concentrations [5,6]. Their biological activity results in antioxidant properties that can be applied as neuro- [7], cardio- [8,9] and radioprotectors [10], as well as anti-inflammatory drugs [11]. Moreover, ceria nanoparticles are capable of exhibiting either anti- or prooxidant properties, depending on the difference in pH levels in various subcellular regions [12]. CeO_2_ can also act as an enzyme mimetic and enhance the action of natural enzymes [13,14]. The exact mechanism of the biological action of ceria is a subject of discussion [10,15], however, it has been shown that the antioxidant activity of CeO_2_ nanoparticles is directly dependent on their size [16].

Cancer cells produce reactive oxygen species more actively than healthy cells due to the lack of redox control [17]. Prooxidant agents cause additional oxidative stress in cancer cells and lead to their apoptotic death [18]. Accordingly, CeO_2_ nanoparticles are more cytotoxic for cancer cells, while the opposite is observed for healthy cells [19]. In addition, due to its pH sensitivity, ceria exhibits vector properties and can target the delivery of radiopharmaceuticals to the affected tissue [20].

Previously, there have been several attempts of using CeO_2_ nanoparticles for nuclear medicine for simultaneous diagnosis and radioprotection [21,22,23]. However, in terms of nuclear-physical characteristics, there are no isotopes of cerium that are optimal for diagnosis or therapy [24]. Ceria nanoparticles have also been used as a part of composite material for ^68^Ge/^68^Ga generator for clinical uses [25]. Another approach for obtaining radionuclide-labeled nanoparticles is indirect conjugation using bifunctional chelators, which bind to the nanoparticle and then form a complex compound with the radionuclide [26]. Modifying the surface of these particles [27] with bifunctional chelators could open new perspectives and expand the number of radionuclides that can potentially be used.

Thus, the study of cerium dioxide conjugates and their complexes with radionuclides of other elements applicable in nuclear medicine is a promising direction for developing combined radiopharmaceuticals with novel properties. These nanoplatforms can then form complex compounds with radionuclides for both diagnostic and radionuclide therapy. The best approach for the latter is the use of alpha-emitters. Due to their relatively low range in the tissue and high linear energy transfer, they minimize damage to healthy organs and tissues [28]. Bismuth radionuclides ^212^Bi (α, T_1/2_ = 60.5 min) and ^213^Bi (α, T_1/2_ = 46 min) have attracted great interest due to their compatible nuclear properties, and some of their compounds have already shown promising results in clinical trials [29,30]. Thus, the conjugation of CeO_2_ conjugates with alpha-emitting ^212^Bi or ^213^Bi could lead to a radiopharmaceutical for radionuclide therapy, which has a radioprotective effect for non-cancer cells.

The most widely used method of radioactive labelling is the modification of the particle surface with a multidentate bifunctional chelator (BFC) followed by the complexation with a radionuclide [26]. The selection of the BFC depends on the radionuclide, the synthesis strategy, physical properties, the desirable polarity and biodistribution properties. Previously, the NPs-azacrown ligand-radionuclide systems for application in radiopharmaceutical purposes have already been studied. For example, the authors of the article [31] tested AuNP modified by peptide-DOTA (1,4,7,10-tetraazacyclododecane-1,4,7,10-tetraacetic acid) conjugates for drug delivery in pancreatic and colon cancer cell lines. In vivo application of these functionalized nanoparticles with proper coating can improve the quality of PET images. Another study [32] reported the use of ^177^Lu labelled conjugates of silica nanoparticles and DOTA for targeted radiation therapy in melanoma models. In addition, it was previously shown that azacrown ethers and their derivatives with the number of heteroatoms in the cycle 5 and 6 form stable complex compounds with many metal cations, including Bi^3+^ [33]. Therefore, these macrocyclic ligands can be used as bifunctional chelators for bonding the radionuclide to nanoparticles.

**Figure 1:**
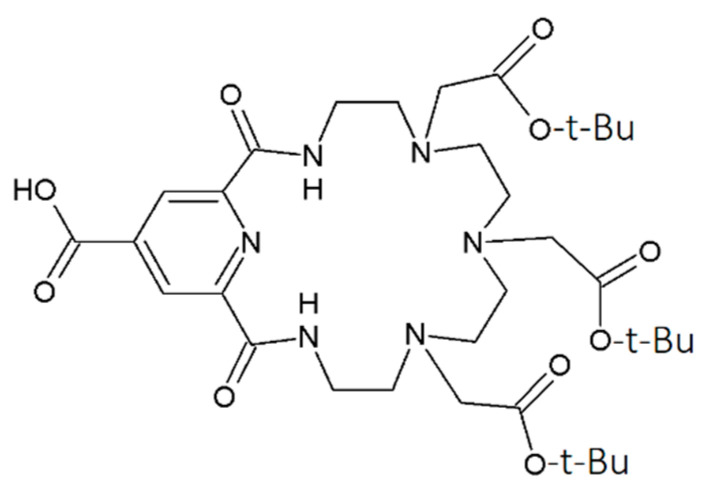
Structure of ligand L investigated in this study.

To the best of our knowledge, this study presents the first ever report of the possibility of modifying ceria nanoparticles with a macrocyclic azacrown ligand for applications in radiopharmaceuticals. The macrocycle with a relatively large cavity (Figure 1) is chosen as the model object since its complexes with bismuth radionuclides have already demonstrated high in vitro and in vivo stability [33].

## 2. Experimental

### 2.1. Reagents

Solutions of cerium ammonium nitrate (NH_4_)_2_Ce(NO_3_)_6_ (Sigma-Aldrich) were used as precursors for the synthesis of cerium dioxide nanoparticles. The chemicals used in this research were chemically pure 2-(chloromethyl)oxirane (ECH) as the linker, 2-(1H-benzotriazol-1-yl)-1,1,3,3-tetramethyluronium hexafluorophosphate (HBTU), aqueous solutions of NaOH and NH_3_, triethylamine, dimethyl sulfoxide (DMSO), 85% formic acid solution, and deionized water (Milli-Q, 18 MΩm). A solution of [^207^Bi]Bi3+ in HCl (0.9 MBq) as a labeling agent was purchased from JSC Ritverc. Bi(ClO_4_)_3_ or BiCl_3_ solutions were used as a carrier for ^207^Bi. Azacrown ligand L (Figure 1) was prepared by a previously reported method [34].

### 2.2. Nanoceria Synthesis and Surface Functionalization

**Figure 2:**
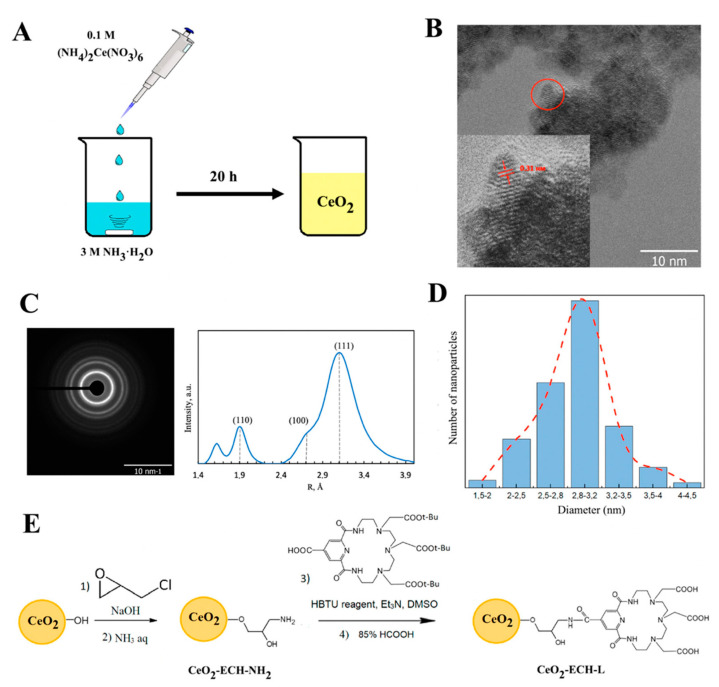
(**A**) Synthesis of CeO_2_ NPs by chemical deposition. (**B**) TEM image of the CeO_2_ surface (scale bar = 10 nm). The inset shows a high-resolution TEM image of the as-prepared CeO_2_ NPs. (**C**) Selected area electron diffraction (SAED) data. (**D**) CeO_2_ NPs size distribution according to TEM data. (**E**) Schematic showing the modification of cerium nanoparticles with azacrown ether L through the ECH linker.

The functionalization of cerium oxide nanoparticles was carried out as follows. The method consisted of the consecutive synthesis of particles (Figure 2A) modified by the linker and ligand L (Figure 2E). Ceria nanoparticles were obtained by chemical deposition from 0.1 M of (NH_4_)_2_Ce(NO_3_)_6_ by 3 M of NH_3_·H_2_O at room temperature and constant stirring for 24 h. As a result, a yellow precipitate of cerium dioxide was formed, which was subsequently decanted by centrifugation, washed with water twice, and dried. In the following stage, 50 mg of the as-prepared CeO_2_ yellow power was suspended in 2 mL of a 0.1 M NaOH solution for 5 min. Then, 1 mL of ECH was added, followed by the addition of 100 µL of 2 M NaOH. The suspension was stirred at ambient conditions for 12 h. The reaction mixture was centrifuged, and the supernatant was decanted. The nanoparticles were washed with water then centrifuged until the pH value of the suspension reached 7. The resulting nanoparticles (1.1 equiv.) were added to a solution of ligand L (1 equiv.), HBTU (3.5 equiv.), and triethylamine (3.5 equiv.) in DMSO and stirred at room temperature for 12 h. Upon completion of the reaction, the precipitate was separated by centrifugation and washed with water. At the final stage, 85% formic acid solution was added and stirred for 3 h at room temperature to remove the tert-butyl protection of the carboxyl groups of the ligand L. The resulting materials were separated, washed with water, and dried.

### 2.3. Characterization of Modified Nanoparticles

*Electron microscopy:* The microstructures of the samples were studied by transmission electron microscopy (TEM) on a Zeiss Libra 200FE electron microscope. The analysis of interplanar spacings was carried out using electron diffraction data obtained during the TEM experiment. The size of particles was calculated by determining the average diameter of approximately 300 particles according to the TEM data. 

*Spectroscopy method:* Infrared (IR) spectra were recorded at 25 °C on a Thermo Scientific Nicolet iS5 FT-IR spectrometer either in KBr or on the working surface of the internal reflectance attachment using a diamond optical element by attenuated total reflection (ATR). The spectral resolution was 4 cm^−1^. Raman spectra were obtained using a Renishaw inVia Raman spectrometer with a 50-mW laser diode at a wavelength of 633 nm. The spectral range was set between 100 and 3500 cm^−1^.

*Thermogravimetry:* The efficiency of ligand binding to the surface of CeO_2_ NPs was analyzed by thermogravimetric analysis (TGA). The measurements were carried out in an atmosphere of air using a Jupiter NETZSCH STA 449 F1 thermal analyzer combined with a quadrupole mass spectrometer NETZSCH QMS 403 C Aëolos, from 30 to 900 °C at a heating rate of 10 °C/min. 

*ζ-potential determination:* The modification of the surface of nanoparticles at each stage was confirmed by determining the isoelectric point from a series of ζ-potential measurements at different pH values in the range 2–11. The pH values of a series of samples in 0.01 M NaClO_4_ were established by adding various concentrations of NaOH or HClO_4_ solutions. The ζ-potential was assessed by dynamic light scattering on a Malvern ZETASIZER nano-ZS instrument. 

*X-ray absorption spectroscopy:* HERFD-XANES experiments were performed at BM20 beamline of the European Synchrotron Radiation Facility, Grenoble (France) [35]. The incident energy was selected using the 〈111〉 reflection from a double crystal Si monochromator. Rejection of higher harmonics was achieved by two Rh mirrors working at an angle of 2.5 mrad relative to the incident beam. HERFD-XAS spectra were measured using an X-ray emission spectrometer [36] at 90° horizontal scattering angle. Sample, analyzer crystal and Si detector (Ketek) were arranged in a vertical Rowland geometry. The Ce HERFD-XAS spectra at the L_3_ edge were obtained by recording the maximum intensity of the Ce Lα1 emission line (4839 eV) as a function of the incident energy. The emission energy was selected using the 〈331〉 reflection of five spherically bent Ge crystal analyzers (with R = 1 m) aligned at an 80.7° Bragg angle. The vertical size of the beam at the sample, which defines the energy resolution, was 80 μm. A combined (incident convoluted with emitted) energy resolution of 1.2 eV was obtained, as determined by measuring the FWHM of the elastic peak. Samples for the HERFD-XANES measurements were prepared as wet pastes and sealed with single kapton confinement (of 25 mm thickness).

### 2.4. Stability in Buffer Solutions

The conjugates of CeO_2_ with ligand L (CeO_2_-ECH-L) were complexed with a solution of [^207^Bi]BiCl_3_ as a long-lived analogue of medically applicable ^212^Bi and ^213^Bi. The stability of the resulting complex CeO_2_-ECH-L-^207^Bi was studied in the following media: 0.9% NaCl, phosphate-buffered saline (PBS) with a pH of 7.4, 0.05 M 4-(2-hydroxyethyl)-1-piperazineethanesulfonic acid (HEPES) and in the fetal bovine serum 1:1 (FBS) (HyClone). Complexes with a solid phase concentration of 10^−3^ g·l^−1^ and a [^207^Bi]Bi^3+^ concentration of 10^−9^ M were prepared in solutions of 0.9% NaCl by the addition of ammonium acetate buffer solution (0.1 M) to fix the pH in the range 6.5–7.2, and in PBS, HEPES or FBS. All samples were incubated at 37 °C and continuously stirred. The labeled CeO_2_-^207^Bi and CeO_2_-ECH-L-^207^Bi were extracted from the mixture by solid–liquid separation behaviors in centrifugal sedimentation. Aliquots of the supernatant were sampled and analyzed using gamma spectrometry.

## 3. Results and Discussion

### 3.1. Synthesis and Functionalization of CeO_2_ Nanoparticles

A schematic of the synthesis of cerium dioxide nanoparticles is given in Figure 2A. According to TEM data (Figure 2B), spherical crystalline nanoparticles were formed using the synthesis procedure. The insert in Figure 2B demonstrates a high-resolution micrograph showing lattice bands of strongly crystallized particles with a distance of 0.31 nm, which corresponds to the (111) plane of CeO_2_ nanoparticles. The electron diffraction (ED) results obtained during the microscopic investigation show that only diffraction reflections corresponding to the CeO_2_ fluorite structure are observed (Figure 2C). The diameter of the particles is 2.9 nm according to the analysis of TEM data (Figure 2D).

The functionalization of CeO_2_ nanoparticles was conducted according to the scheme in Figure 2E. Typical TEM images and particle size distribution of the surface-modified nanoceria are shown in Figure S1 in the Supplementary Information. The average diameter of CeO_2_-ECH-L is 2.8 nm with a standard deviation of 0.2 nm. Nanoparticles of the obtained conjugates had a hydrodynamic size of 10–17 nm according to DLS results (see Figure S2 in the Supplementary Information). This size did not change in buffer solutions (HEPES and PBS) as well as in saline.

**Figure 3:**
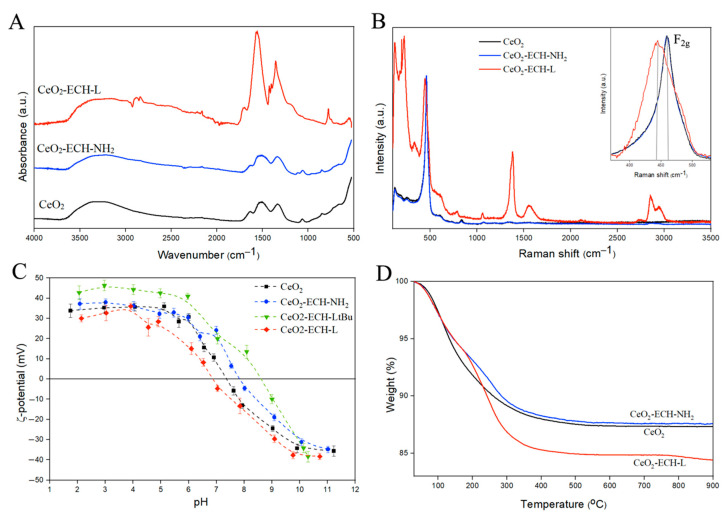
(**A**) IR spectra of the initial CeO_2_ and synthesized functionalized CeO_2_ nanoparticles. (**B**) Raman spectra obtained from samples of the initial CeO_2_ and modified series. The inset shows enlarged Raman spectra, where the change in the position of the F_2g_ peak (465 cm^−1^) of the modified samples relative to the comparison sample CeO_2_ is observed. (**C**) Dependence of the zeta potential on the pH value (I = 0.01 M). (**D**) weight loss curves obtained from thermogravimetric analysis.

The prepared nanoparticles were pre-modified with epichlorohydrin (ECH) to further obtain a hydrophilic surface coated with amino groups. At the next stage, ligand functionalization was carried out through an unprotected carboxyl group by the method of peptide synthesis, followed by the removal of tert-butyl protective groups to obtain the CeO_2_-ECH-L conjugate. Infrared (Figure 3A) and Raman (Figure 3B,C) spectroscopy was used to determine the structure and composition of the materials and functionalization effectiveness. The deformation Ce-O bands (845, 940, 1060, 1330 cm^−1^) are observed in the IR spectra for all samples. Vibrations of R-NH_2_ groups in the region of 1100 cm^−1^ confirm the formation of the CeO_2_-ECH-NH_2_ structure. After ligand L functionalization, peaks characteristic of the amino group (3500–3300, 1110 cm^−1^), the formed secondary amide (1694 cm^−1^), vibrations in the pyridine ring (1567–1557, 778 cm^−1^), and carboxyl groups (1425, 1403 cm^−1^) of the organic molecules are traced (Figure 3A).

A clear difference between the analyzed samples can be observed by Raman spectroscopy. Figure 3B shows the Raman spectra of the synthesized functionalized CeO_2_ nanoparticles. The spectra were recorded at minimum laser intensities (50 mW) to avoid a possible degradation effect of the samples. The first order of the CeO_2_ Raman spectrum is characterized by the presence of a F_2g_ vibrational mode at approximately 465 cm^−1^, associated with the elongation of the Ce–O bond, where Ce and O are coordinated at eight and four times, respectively. The weak band observed at 1050 cm^−1^ may be related to the asymmetry of the primary A_1g_ mode, combined with small additional contributions of E_g_ and F_2g_ symmetries. The presence of a band in the modified CeO_2_ samples at approximately 600 cm^−1^ can be observed, which may be related to the presence of Ce^3+^ on the surface of the solid, promoting a non-stoichiometric condition and the appearance of this band at 600 cm^−1^. Moreover, the presence of bond vibrations in the Raman spectra of organic molecules confirms the change in the structure of the surface of cerium dioxide nanoparticles [37,38].

The bands in the Raman spectra can shift with changes on the surface of the crystal [39]. The change in the half-width at half-maximum and the shift of the maximum peak at 465 cm^−1^ (F_2g_) of the modified series of particles relative to the initial CeO_2_ are shown in Figure 3C. Based on the above observation, the surface of the nanoparticles has changed, which confirms the change in the structure of the substances.

The modification of the surface of nanoparticles at each stage was confirmed by a series of ζ-potential measurements at different pH values, as shown in Figure 3D. The functionalization of particles by amino groups on the CeO_2_ surface show a shift of the isoelectric point in the region with a high pH value (pI = 7.4). The shift of the isoelectric point to the left for CeO_2_-ECH-L-tBu (pI = 6.8) is explained by the presence of carboxyl groups in the ligand structure, which are deprotonated with increasing pH value, forming a negative charge on the surface. At the same time, nitrogen atoms in the macrocyclic and pyridine fragments of the ligands also affect the pI position. They shift the isoelectric point to the region with low pH values due to their basic properties. Thus, from a combination of IR and Raman spectroscopy and ζ-potential results, we can conclude that CeO_2_ nanoparticles were functionalized with azacrown ether L.

The effectiveness of ligand binding to the surface of ceria nanoparticles was evaluated using the method of thermogravimetric analysis combined with mass spectrometry (TGA-MS) in an atmosphere of air (Figure 3D). Weight loss occurs smoothly in the range from 30 °C to 400 °C. The weight loss for CeO_2_-ECH-L is greater compared to CeO_2_, which is a consequence of ligand presence on the surface. In addition, there is a change in the tilt angle of the lines of the modified sample at the beginning of heating caused by the oxidation of the organic part of the material. The TGA spectra of the free ligand are shown in Figure S3 in the Supplementary Information. According to the TGA-MS results, the release of water for CeO_2_ and CeO_2_-ECH-NH_2_ NPs begins from the moment of heating and ends at temperatures up to 200 °C due to the presence of a substantial amount of water on the surface of the sample (see Figure S4 in the Supplementary Information). For conjugation of the nanoparticles and ligand L, water is released when heated to 400 °C due to the oxidation of the organic molecules on the particles. The insignificant amount of CO_2_ registered for CeO_2_ is related to experimental conditions during particle synthesis. In other cases, the CO_2_ peak in the mass spectra confirms the presence of organic molecules on the CeO_2_ surface.

**Figure 4:**
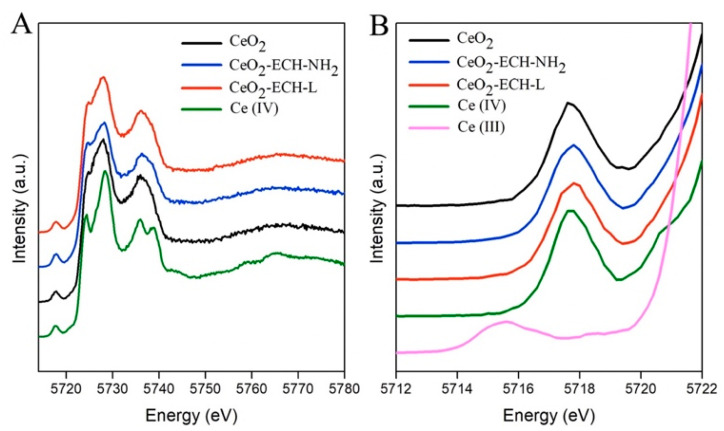
(**A**) Ce L_3_ edge HERFD-XANES data for CeO_2_ nanoparticles before and after surface modification, compared to CeO_2_ and Ce_2_(SO_4_)_3_, as Ce(IV) and Ce(III) references, respectively. (**B**) Enlarged pre-edge region.

Figure 4 shows the HERFD-XANES data for the investigated samples. CeO_2_ (NIST) was used as a standard with a cell parameter of 5.4117 Å and a particle size of more than 25 nm. 

The HERFD-XANES spectra reveal the presence of the dipole-allowed 2p-5d transitions (main edge transitions in the energy range 5723–5730 eV) and of the dipole forbidden but quadrupole-allowed 2p-4f transitions (at the pre-edge in the energy range 5715–5720 eV) [40,41]. The spectra of the studied samples relative to the standard in the main edge region have a smoother shape due to the size effect. This effect was discussed earlier by Plakhova et al. [15]. The presence of Ce (III) would lead to the appearance of an additional peak in the pre-edge (~5715.5 eV) due to an increase in the number of electrons and electron–electron interactions on the 4f orbital. The shape and position of the pre-edge peaks (~5717.8 eV) of our CeO_2_ samples before and after the modification process corresponds to the Ce (IV) standard (Figure 4B). Thus, the modification of the CeO_2_ surface with azacrown ether ligand does not change the cerium oxidation state in nanoparticles, despite the use of reducing agents in the synthesis procedure.

### 3.2. Stability in Buffer Solutions

The stability of the CeO_2_-ECH-L-^207^Bi nanoplatform and the extent of bismuth absorption on the surface of nanoparticles were studied in 0.9% NaCl with ammonium acetate buffer solution, PBS with pH = 7.4, and HEPES (pH 7.1). Dissociation of the complex under these conditions is a highly probable process due to the large number of competing ions capable of re-chelating radionuclide cations. This experiment can partially simulate the behavior of a potential radiopharmaceutical when it is injected into a living organism.

**Figure 5:**
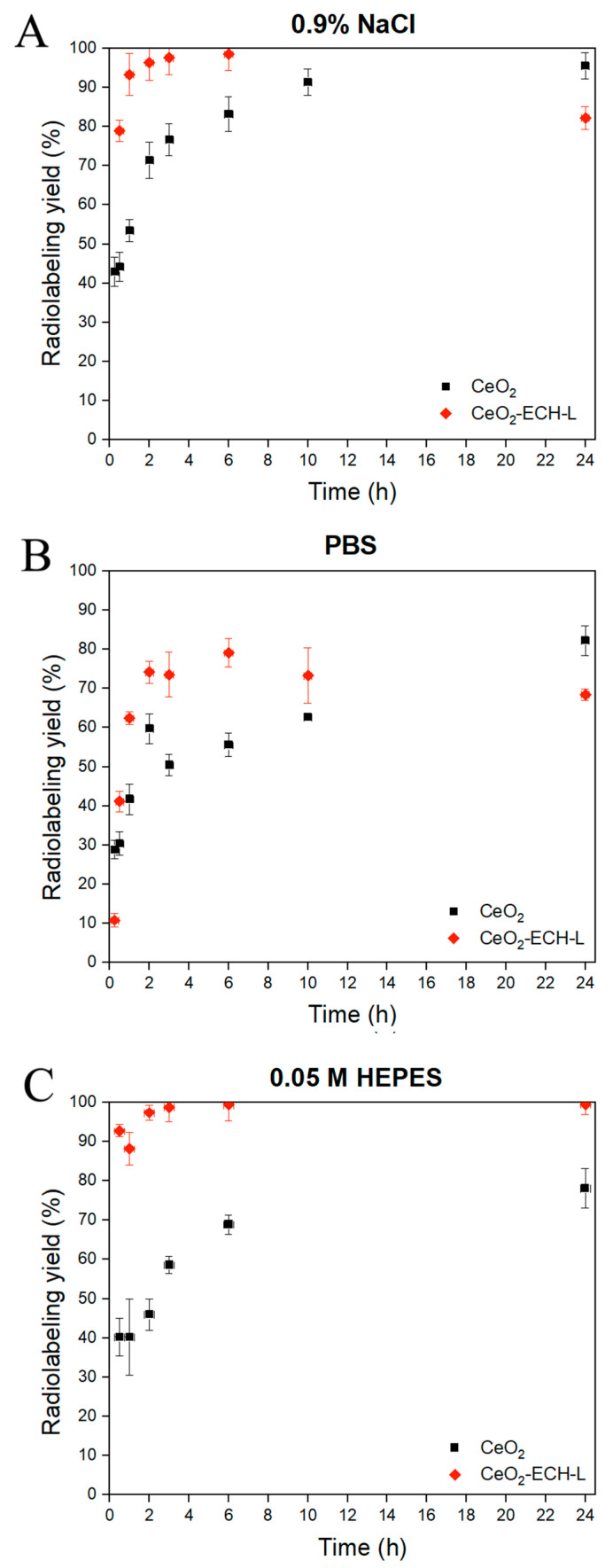
Kinetic stability of complexes CeO_2_-^207^Bi and CeO_2_-ECH-L-207Bi in buffer solutions: (**A**) 0.9 % NaCl with ammonium acetate buffer solution (pH = 6.8); (**B**) PBS (pH = 7.4); (**C**) 0.05 M HEPES (pH = 7.1).

Figure 5A–C shows the labeling efficiency at different time intervals of equilibration under constant stirring and at a temperature of 37 °C. In Figure 5A,B, the conjugate of the nanoparticles and ligand bonds with the radionuclide ^207^Bi over 90% after 30 min of mixing, and after 4 h the radiolabeling yield is quantitative. The formation of nanoparticle-ligand-radionuclide structures is relatively slow, which could be due to the presence of competing ions in the solution. Moreover, as a result of re-chelation of [^207^Bi]Bi^3+^ in the solution, the radiolabeling yield does not exceed 80% in this biological media.

As shown in Figure 5C, [^207^Bi]Bi^3+^ binds rapidly to the ligand L on the surface of the nanoceria in 0.05 M HEPES, and after 30 min, the radiolabeling yield exceeds 90%. During the day, the complex CeO_2_-ECH-L-^207^Bi does not dissociate and the yield is c.a. 99% after 24 h. At the same time, the absorption of bismuth cations on CeO_2_ reaches 75% after 24 h, however, this process exhibits slow kinetics compared with the formation of the complex. This fact can be explained by the absence of re-chelating agents in the buffer. The high kinetic stability of the conjugate of nanoparticles and the ligand L with radionuclide ^207^Bi is obtained in HEPES, which makes it possible to use this system for in vivo experiments.

**Figure 6:**
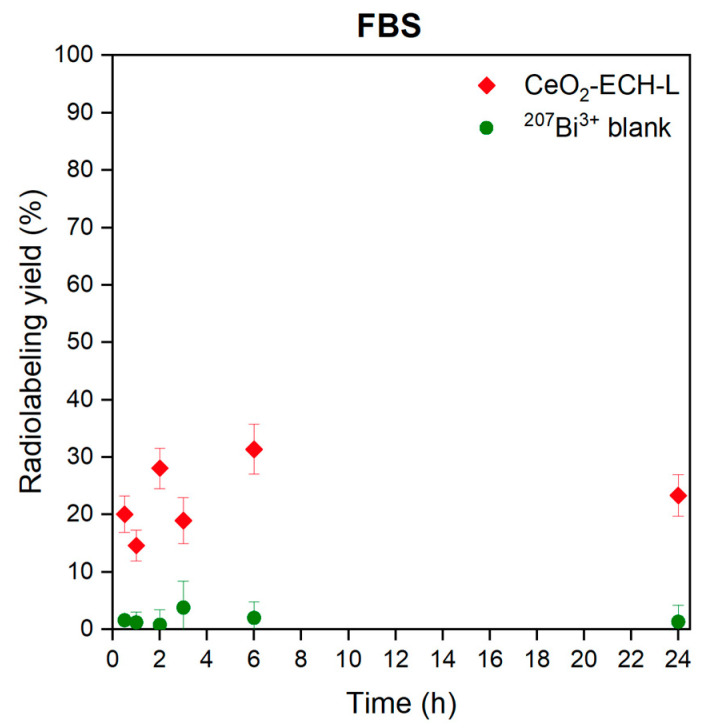
Dependencies characterizing the kinetic stability of CeO_2_-ECH-L-^207^Bi and ^207^Bi^3+^ in fetal bovine serum (FBS) in a ratio of 1:1.

The stability of the CeO_2_-ECH-L-^207^Bi was analyzed in the FBS. This experiment can partially simulate the behavior of a potential radiopharmaceutical when it is introduced into a living organism and then circulated in the blood. As shown in Figure 6, the radiolabeling yield does not exceed 30% after 24 h for CeO_2_-ECH-L-^207^Bi. Dissociation of the complex under these conditions is a highly probable process due to the large number of serum proteins that are able to re-chelate radionuclide cations. 

We can conclude that the results of kinetic stability in FBS during the day for CeO_2_-ECH-L-^207^Bi are significantly lower than acceptable values for radiopharmaceuticals. On the other hand, the conjugate CeO_2_-EPC-L can potentially be used for labelling with other radionuclides that demonstrate the stability of complexes under in vitro and in vivo conditions.

## 4. Conclusions

This study proved that CeO_2_ nanoparticles are promising nanoagents in nuclear medicine. The functionalization of nanocerium by a macrocyclic azacrown ligand with six heteroatoms in the cavity and three pendant carboxylic arms is shown. The resulting conjugate forms a radiolabeled complex with ^207^Bi^3+^, which is stable in saline and HEPES solutions but dissociates into FBS. These results are promising for further studies of the possible applications of this conjugate as a radiopharmaceutical. Moreover, we believe that the proposed method of conjugation can be extended to other azacrown ethers and CeO2–ECH-L conjugate can be used as a multipurpose nanoplatform with various radionuclides for molecular imaging, therapy and theranostics of oncological diseases.

## Data Availability

Not applicable.

## Supplementary Materials

The following supporting information can be downloaded at: https://www.mdpi.com/article/10.3390/nano12244484/s1, Figure S1: TEM image of the CeO_2_-ECH-L surface (scale bar = 50 nm) and CeO_2_-ECH-L size distribution according to TEM data; Figure S2: CeO_2_-ECH-L size distribution in the different buffer solutions according to DLS data; Figure S3: Curves obtained from thermogravimetric analysis of the CeO_2_ and synthesized functionalized CeO_2_ nanoparticles; Figure S4: TGA-MS results of initial (A) and surface-modified CeO_2_ nanoparticles (B,C) obtained for the mass numbers 18 (H_2_O) and 44 (CO_2_).
