# Supplementary material for: CeO2-Azacrown Conjugate as a Nanoplatform for Combined Radiopharmaceuticals
Source: Nanomaterials (Basel). 2022 Dec 18;12(24):4484. doi: 10.3390/nano12244484 (PMC9783244; doi:10.3390/nano12244484)
Supplement: Supplementary file 1 [file nanomaterials-12-04484-s001.zip › nanomaterials-1869183-Supplementary.pdf]

CeO_2-azacrown conjugate as a nanoplatform for combined radiopharmaceuticals

Supplementary Information

Sofia Khabirova [1,], Gleb Aleshin [1], Tatiana Plakhova [1], Anastasia Zubenko [2], Anna Shchukina [2], Olga Fedorova [2], Aleksey Averin [3], Ekaterina Belova [1], Elena Bazarkina [4,5], Kristina Kvashnina [1,4,5] and Stepan Kalmykov [1]*

[1] Department of Chemistry, Lomonosov Moscow State University, Leninskie Gory, 1/3, 119991, Moscow, Russia
[2] N. Nesmeyanov Institute of Organoelement Compounds of Russian Academy of Sciences, Vavilova, 28, GSP-1, 119991, Moscow, Russia
[3] Frumkin Institute of Physical Chemistry and Electrochemistry Russian Academy of Sciences, Leninskiy Ave. 31b4, 119991, Moscow, Russia
[4] The Rossendorf Beamline at ESRF—The European Synchrotron, CS40220, CEDEX 9, 38043 Grenoble, France
[5] Helmholtz Zentrum Dresden-Rossendorf (HZDR), Institute of Resource Ecology, P.O. Box 510119, 01314 Dresden, Germany
* Correspondence: khabirovasophia@gmail.com; Tel.: +7-9661919972

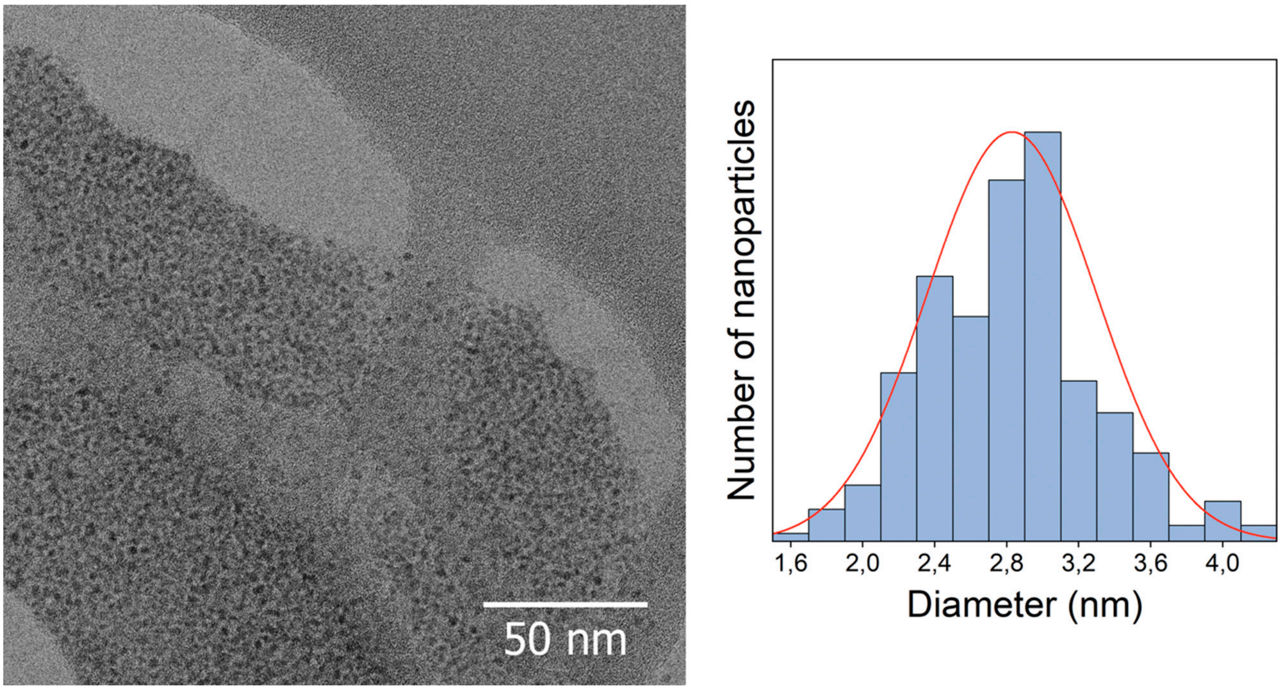

Figure S1. TEM image of the CeO_2-ECH-L surface (scale bar = 50 nm) and CeO_2-ECH-L size distribution according to TEM data.

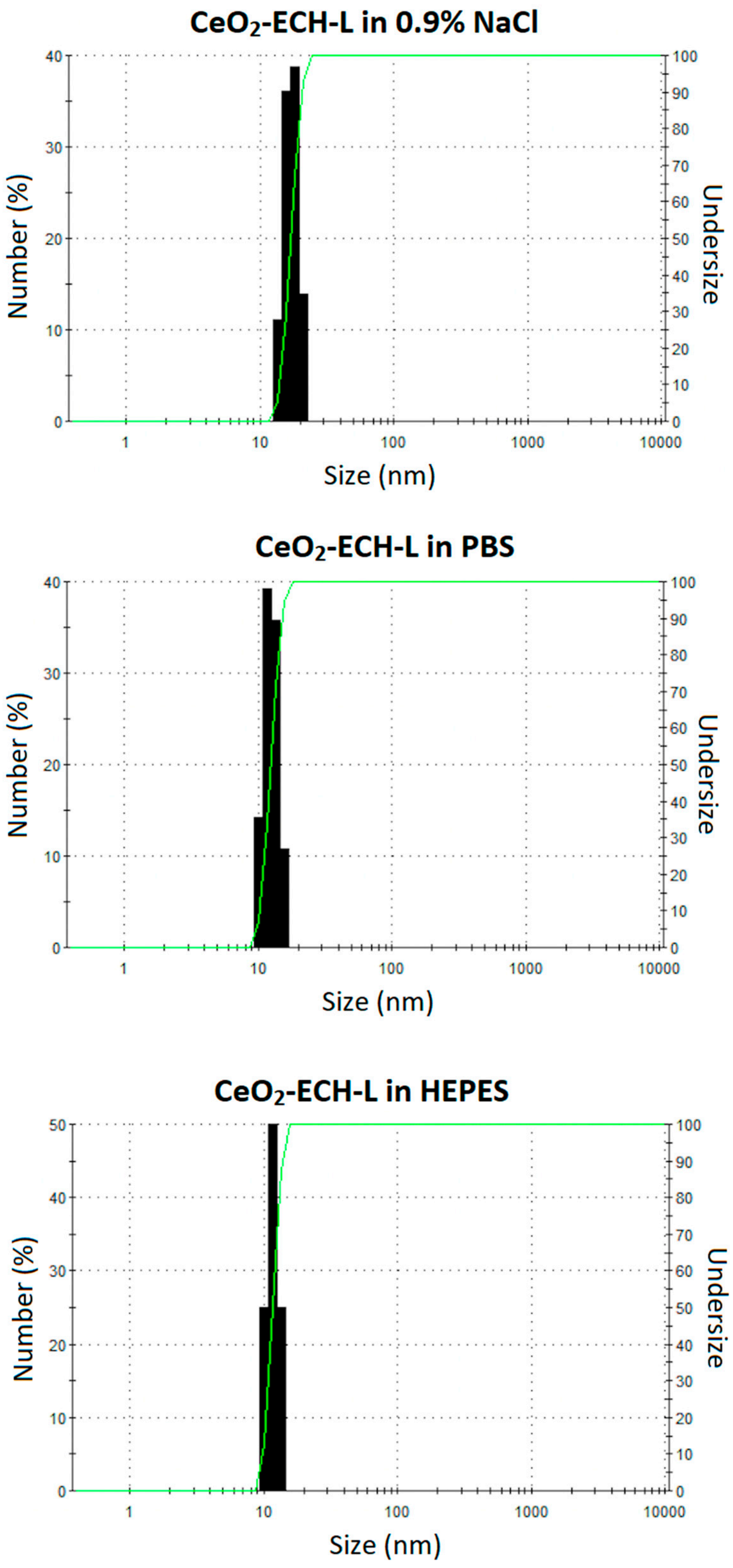

Figure S2. CeO_2-ECH-L size distribution in the different buffer solutions according to DLS data.

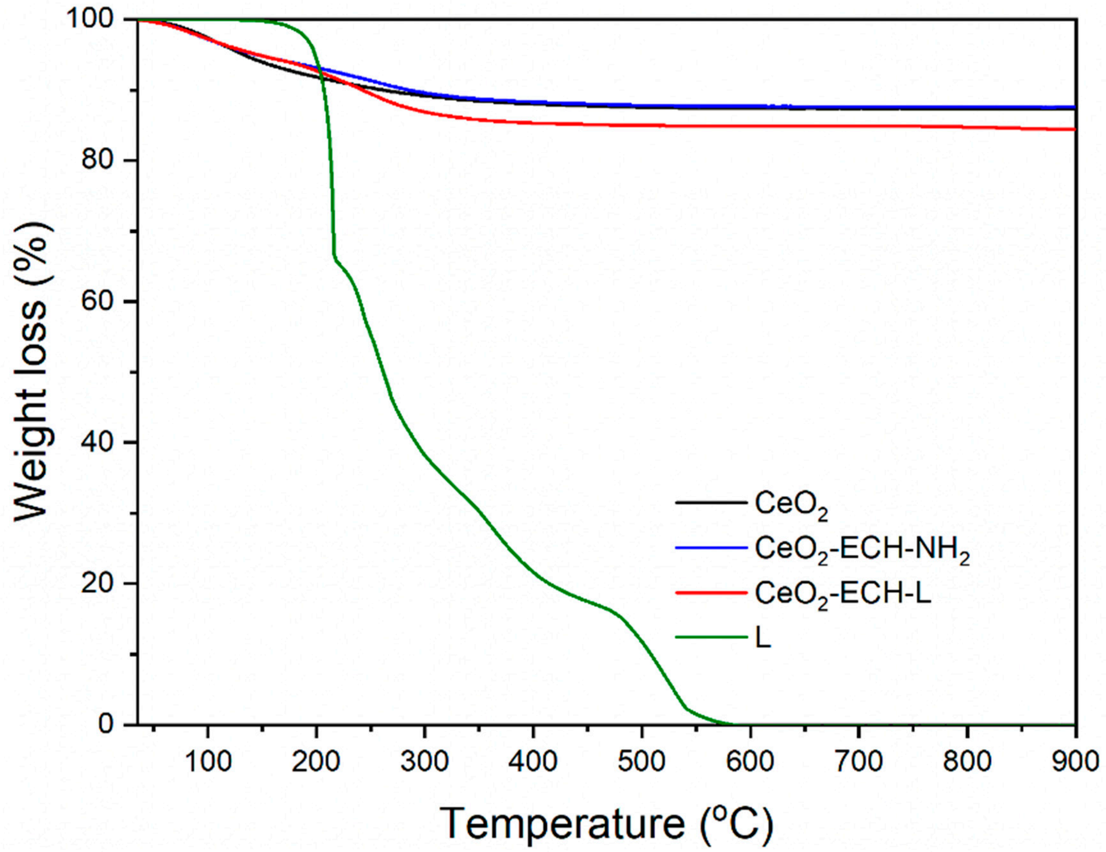

Figure S3. Curves obtained from thermogravimetric analysis of the CeO_2 and synthesized functionalized CeO_2 nanoparticles.

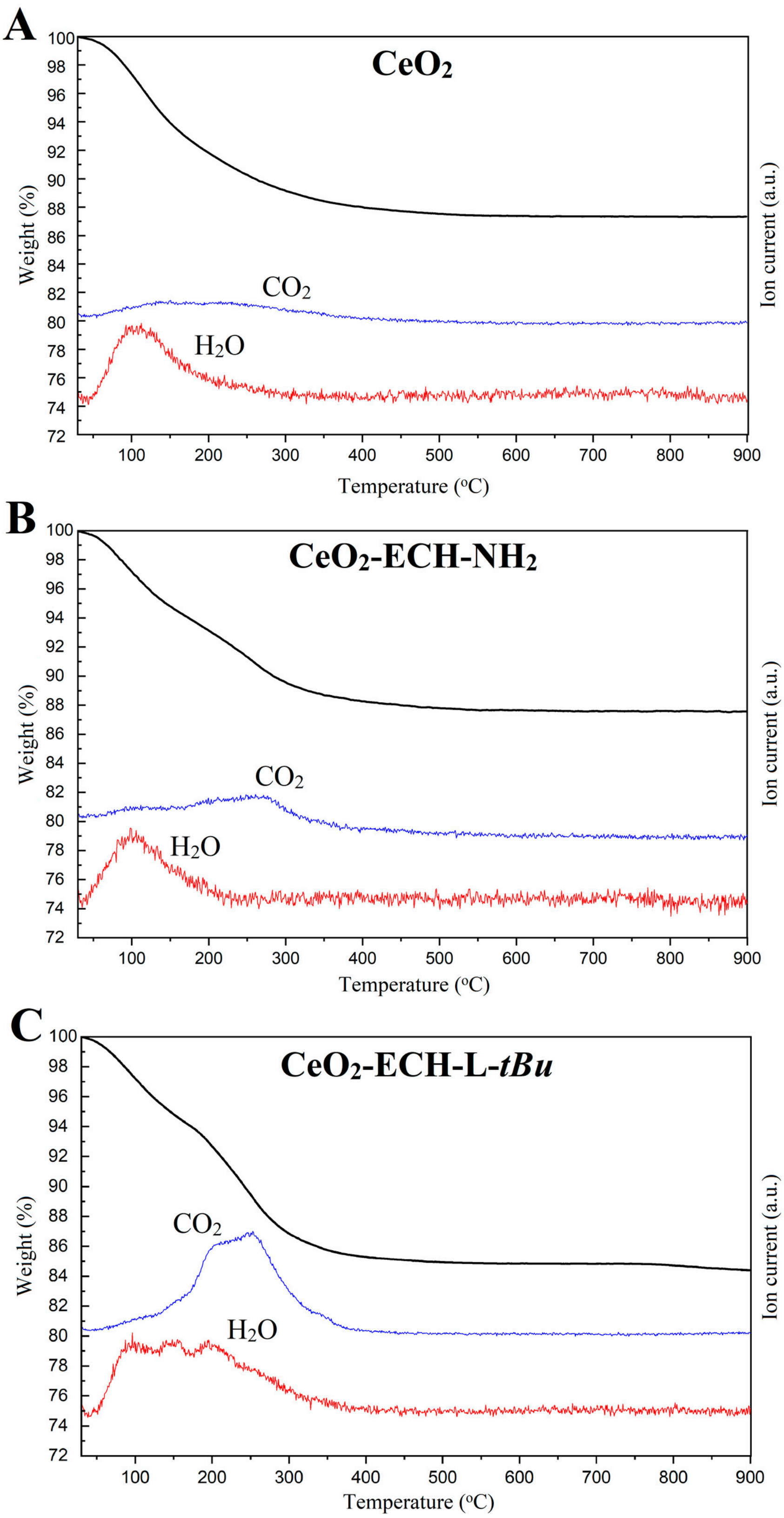

Figure S4. TGA-MS results of initial (A) and surface-modified CeO_2 nanoparticles (B, C) obtained for the mass numbers 18 (H_2O) and 44 (CO_2).
